# Life on the Edge: Ecological Genetics of a High Arctic Insect Species and Its Circumpolar Counterpart

**DOI:** 10.3390/insects10120427

**Published:** 2019-11-26

**Authors:** Jean-Christophe Simon, Frédérique Mahéo, Lucie Mieuzet, Christelle Buchard, Jean-Pierre Gauthier, Damien Maurice, Joël Bonhomme, Yannick Outreman, Maurice Hullé

**Affiliations:** 1 INRA, IGEPP UMR 1349 INRA/Agrocampus Ouest/Université Rennes 1, Domaine de la Motte 35650 Le Rheu and 65, rue de Saint Brieuc, 35000 Rennes, France; frederique.maheo@inra.fr (F.M.); lucie.mieuzet@inra.fr (L.M.); christelle.buchard@inra.fr (C.B.); jean-pierre.gauthier@inra.fr (J.-P.G.); u.bonhomme@wanadoo.fr (J.B.); yannick.outreman@agrocampus-ouest.fr (Y.O.); maurice.hulle@inra.fr (M.H.); 2 INRA, UMR EEF INRA/Université de Nancy, 54282 Champenoux, France; damien.maurice@inra.fr

**Keywords:** *Acyrthosiphon svalbardicum*, *A. brevicorne*, aphids, population genetics, arctic, colonization, adaptation

## Abstract

Arctic ecosystems are subjected to strong environmental constraints that prevent both the colonization and development of many organisms. In Svalbard, few aphid species have established permanent populations. These high arctic aphid species have developed peculiar life-history traits such as shortened life cycles and reduced dispersal capacities. Here, we present data on the distribution and population genetics of *Acyrthosiphon svalbardicum* in Spitsbergen, the main island of the Svalbard archipelago, and compared its genetic structure with that of its close relative *Acyrthosiphon brevicorne,* sampled in the top of Scandinavian mainland. We found that *A. svalbardicum* is common but heterogeneously distributed along the west coast of Spitsbergen. We recorded this species up to 79°12’, which constitutes the northernmost location for any aphid. Genetic structure examined using microsatellite markers showed more pronounced spatial differentiation in *A. svalbardicum* than in *A. brevicorne* populations, presumably due to reduced dispersal capacities in the former species. Although populations of *A. brevicorne* and *A. svalbardicum* were well-delineated at nuclear loci, they shared similar cytoplasmic DNA haplotypes as revealed by sequence analysis of two DNA barcodes. These results raise questions about whether these two taxa are different species, and the colonization sources and history of the Svalbard archipelago by *A. svalbardicum*.

## 1. Introduction

Species are all limited in their geographical distribution. In some cases, geographic ranges are restricted to small areas and are stable over long periods of time, while in others, they include large territories and shift very frequently [1]. Many hypotheses have been proposed for explaining geographic range limits of species and their variation, with special attention to dispersal capacities, species interactions, and physiological constraints. However, such factors usually act in combination to a situation that adds difficulties to understanding why species fail to continue to spread at some points [2]. Furthermore, the population genetics at the edge of a species’ range and its consequences for evolutionary dynamics remain poorly known, although important advances have been made recently on invasive organisms [3]. In this context, the Arctic environment constitutes an interesting place to study range limits of species because of marked ecological conditions and reduced species richness compared with more temperate latitudes [4]. This is the case for the Arctic insect fauna, which is considerably diminished, especially on Arctic islands because of exacerbated dispersal limitation.

Spitsbergen (70° to 80° North) is the main island of the Svalbard archipelago and is about 700 km from the top of the Scandinavian mainland. The climate of this archipelago is characterised by sub-zero temperatures for most of the year, and only a short summer season allows the growth and reproduction of arthropods [5]. The harsh conditions associated with insularity constitute big challenges for the establishment of new species on Svalbard. Unlike gradual adaptations across environmental gradients, successful colonization of arctic islands requires abrupt and rapid physiological and ecological adjustments to cope with the reduced energy budget and novel biotic interactions [4,5]. Consequently, only a few long-distance colonization events may lead to population establishment [6]. The Svalbard archipelago is therefore a good ecosystem in which to study how geographic and environmental barriers in colonization and establishment shape species diversity, spatial distribution and population structure. To date, few studies have been conducted on the population genetics of arctic organisms such as plants and vertebrates, and none of them concern insects.

Among the 230 insect species reported on Svalbard [7], only three aphid species are resident on Spitsbergen: *Acyrthosiphon svalbardicum,* which feeds exclusively on *Dryas octapetala* (Rosaceae)*, Sitobion calvulum*, which feeds primarily on *Salix polaris* (Salicaceae)*,* and a *Pemphigus* sp., which reportedly feeds on roots of *Poa* spp. (Poaceae) [5]. Of these three species, *A. svalbardicum* is the most common and has received the most attention. Compared to aphids in temperate zones, this species has a simplified life cycle and a short activity period [8,9]. Typically, parthenogenetic (clonal) females hatch from overwintering eggs by mid-June and give birth directly to sexual females and males. Eggs are laid following mating during mid-July to the beginning of August and overwinter until the following summer. In some instances, *A. svalbardicum* can produce an extra generation, although there are uncertainties whether this three-generation life-cycle is achieved in the field [8,9]. Populations of *A. svalbardicum* are patchily distributed [10] and winged individuals were unknown until the discovery of one alate female in 2001 [11] and several specimens in 2006 in restricted areas of Spitsbergen [12].

*Acyrthosiphon svalbardicum* has a close related species, *A. brevicorne*, recorded at the top of Scandinavia, Greenland and arctic Canada [13], and differing by the presence of abdominal pigmentation [13,14,15]. Although this circumpolar aphid species shares the same host plant (*D. octopetala*) with *A. svalbardicum*, it displays several important biological differences with the latter [16]. Firstly, the number of generations over its annual life-cycle is controlled by photoperiod (while pre-determined in *A. svalbardicum*). Secondly, sexual morph production increases gradually across generations, with several generations in a year (as opposed to *A. svalbardicum,* which typically produces sexual forms only, in the second generation). Thirdly, winged forms are regularly produced and induced by crowding (while occurrence of winged forms is very rare in *A. svalbardicum* and seemingly not related to aphid density).

The main aim of the present study was to examine how the specific life-style of these two species influences their respective population genetic structure. For that, *A. brevicorne* populations were sampled in the top of Scandinavia during the summers of 2006, 2010, and 2012, while *A. svalbardicum* populations were intensively surveyed along the west coast of Spitsbergen during the summers of 2004, 2005, 2006, and 2009. The genetic structure of these various population samples was analysed with microsatellite markers as described here. Considering the biological differences between these two aphid species, we expected higher rates of inbreeding and stronger spatial differentiation in *A. svalbardicum* populations compared with *A. brevicorne*. Due to the small morphological differences between the two aphids, we also measured the molecular divergence between *A. brevicorne* and *A. svalbardicum* by analysing two DNA barcodes in an attempt to clarify their taxonomic status. From this combined approach, we could refine the geographic distribution of *A. svalbardicum* on Spitsbergen, highlight the impact of environmental constraints on the population genetics of two polar aphids with contrasting life-styles and distributions, and thereby propose a scenario with regard to the colonization history of the Svalbard archipelago by *A. svalbardicum.*

## 2. Materials and Methods

### 2.1. Aphid Sampling

**Figure 1:**
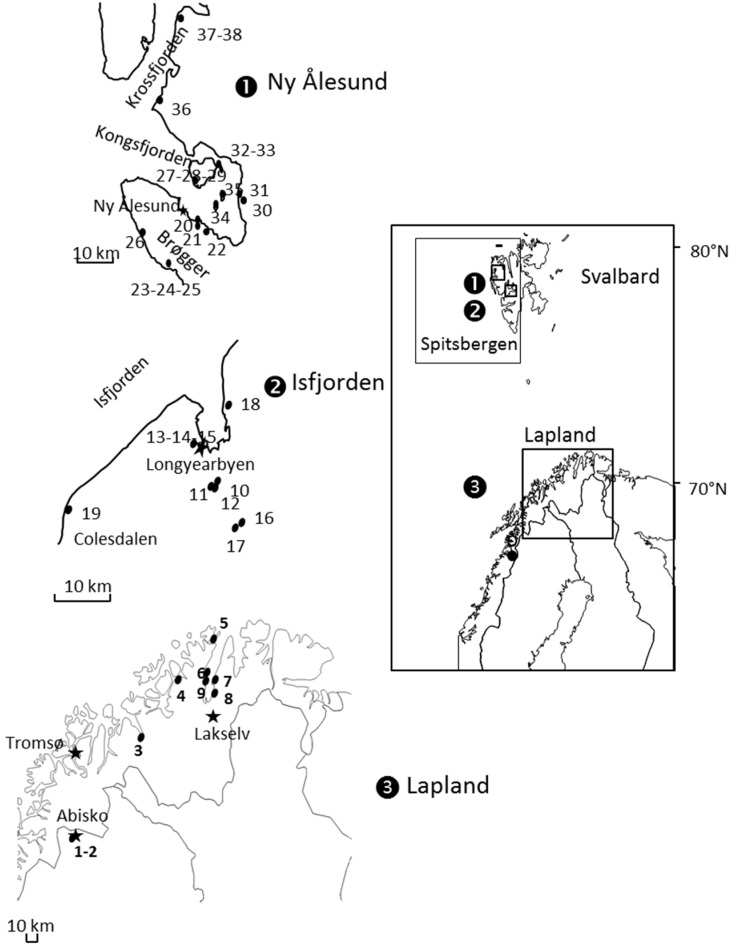
Maps showing the locations of *A. svalbardicum* (zones 1 and 2) and *A. brevicorne* (zone 3) populations sampled for genetic analyses. Only sites where aphids were found are indicated. Stars represent towns or settlements.

Individuals of *A. svalbardicum* were sampled along the west coast of Spitsbergen on mountain avens *Dryas octopetala*, their only host-plant. Aphids were searched by shaking *Dryas* plants under a white tray. Surveys were conducted in July 2004, 2005, 2006, and 2009 in the vicinity of two main zones: Longyearbyen (78°13’N, 15°37′ E, mean annual temperature −5.6 °C) in Isfjorden, and Ny Ålesund (78°55’N, 11°56′ E, mean annual temperature −5.2 °C) in Kongsfjorden (Figure 1). Aphids were also searched with no success in the areas of Barentsburg (78°04′ N, 14°13′ E), Prins Karls Forland (78.55′ N 11.017′ E) and Magdalenefjorden 79°34′ N, 10°58′ E). At each site, where aphids occurred, the geographic position was recorded and about 30 individuals were sampled. Some populations were sampled at different years to assess temporal variation in the genetic structure. In total, 29 samples of *A. svalbardicum* were analysed for genetic structure. At the top of the Scandinavian mainland, we also collected nine populations of the close related species *A. brevicorne* on *D. octopetala* as with *A. svalbardicum*. Two were collected in June 2006 and 2010 at the Abisko Scientific Research Station, Sweden (68°21′ N, 18°49′ E, mean annual temperature 0.2 °C), and seven were collected at various sites in Northern Norway, between Storslett (mean annual temperature 0.1 °C) and Lakselv (mean annual temperature −1.4 °C), in 2012 (Figure 1, Table 1). These samples are referred hereafter to as “Lapland populations”.

### 2.2. Microsatellite Isolation and Aphid Genotyping

Microsatellite markers were isolated from *A. svalbardicum* (and subsequently used for examining population structure on both *A. brevicorne* and *A. svalbardicum*) using a high-throughput method based on coupling multiplex microsatellite enrichment and next-generation sequencing on 454 GS-FLX Titanium platforms [17]. The open access bioinformatics tool QDD [18] was used to analyse the 454 sequences and design primers for amplification of the detected microsatellite motifs. A set of 48 microsatellite loci was first selected from 521 validated by QDD based on the following criteria: perfect motifs, high number of repeats, dinucleotide motifs, Tm comprised between 59 °C and 61 °C, GC percentage between 45 and 55, length of PCR products. This set was thereafter used on a subsample of the *A. svalbardicum* population collected to retain at the end 8 loci showing high polymorphism and alleles easy to score (Table 2).

We used the M13-tailed primer method [19] to label amplicons for visualization on the capillary sequencer. Forward primers were 5′-tailed with a 23-basepair M13 sequence. Loci were amplified in a final volume of 10 µL for polymerase chain reaction (PCR) amplification. The reaction mixture contained 2 µL of template DNA, 0.25 µM of each primer, 0.2 mM of a four nucleotide mixture, 1.25 mM of MgCl_2_, 0.25 µM of 1 µL of PCR Buffer (Promega, Madison, WI, USA) and 0.25 U of Taq DNA Polymerase (Promega, Madison, USA). The M13 primers were5′-fluorescently tagged with HEX, 6-FAM, NED or VIC at 0.25 µM for assessment of allele sizes on a capillary sequencer (describe below). PCR were conducted on S1000 thermal cycler (2008, Bio-Rad Laboratories, Hercules, CA, USA) using the following cycling conditions: initial denaturation at 94 °C for 5 minutes, first cycle of DNA amplification (repeated 20 times) with a denaturation step at 94 °C for 20 seconds, hybridization at 55 °C for 20 s, elongation at 72 °C for 30 s; second cycle of M13 amplification with 20 repetitions of the following steps: 94 °C for 20 s, 53 °C for 20 s and 72 °C for 30 s, and with a final elongation at 72 °C for 5 min.

Diluted PCR products (1.2 μL on 10 μL water) were added to 10 μL of Hi-Di formamide containing 0.7% of 500 LIZ DNA ladder (Applied Biosystems, Foster City, CA, USA) and electrophoresis was performed in the capillary sequencer ABI 3730 (Applied Biosystems, Foster City, CA, USA). Allele sizes were automatically assigned by GeneMapper (version 3.7, Applera Corp., Norwalk, CT, USA) and visually checked.

### 2.3. Genetic Analysis

Within-population genetic diversity: Genotypic diversity within each population sample was assessed using the G/N ratio (where G corresponds to the number of multilocus genotypes and N to the sample size). Mean number of alleles and both observed and expected heterozygosities were calculated using GENEPOP [20]. F_IS_ values were calculated globally and for each locus within each cluster using the software GENETIX v.4.05.2 [21]. Ninety-five per cent confidence intervals of F_IS_ were obtained by bootstrapping individuals 1000 times. Departures from Hardy-Weinberg equilibrium and linkage disequilibrium (LD) computed using the Markov Chain probability test were also assessed using GENEPOP. These within-population genetic estimates were compared between the two aphid species using Linear Mixed Models (i.e., LMMs) assuming a Gaussian error. As some populations were sampled at the same sites but at different time intervals, the site was considered as a random factor in models in order to include temporal dependence. In each LMM, the significance of the species effect was determined using a likelihood ratio test. Model assumptions were verified by plotting residuals versus fitted values for all models and by checking residuals normality. All statistics were performed by using the package lme4 [22] in the software R [23].

Genetic differentiation between populations: Overall and pairwise F_ST_ values were computed with FSTAT 2.9.3.2 [24] and used to quantify differentiation among populations in allele frequencies. Genetic distance between populations was calculated using the Allele Shared Distance [25]. The possibility for an isolation-by-distance pattern was tested by using the Isolde option of GENEPOP, which computes a regression of F_ST_/(1- F_ST_) estimates to geographic distances and performs a Mantel test using a permutation procedure [26].

We also used the Bayesian statistical approach implemented in the program STRUCTURE 2.3.3. [27] to estimate the number of clusters represented by the entire data set, without a priori. Ten iterations were performed using a ‘burning period’ of 50,000 iterations with a 50,000 Markov Chain Monte Carlo (MCMC) step, in admixture models with correlated allele frequencies. The analyses were made for a number of cluster K varying from 2 to 30. The most probable number of clusters was defined using both log probabilities [Pr(X ǀ K)] and ΔK, as described in [28].

### 2.4. Molecular Divergence between A. brevicorne and A. svalbardicum

Since the taxonomic description of *A. brevicorne* and *A. svalbardicum* species has been made on a few samples and the distinction of these two taxa relies mainly on one criterion based on pigmentation [13,14,15], we used a barcoding approach to assess the molecular divergence between several aphid specimens collected from Svalbard (10 individuals from 4 sites), putatively belonging to *A. svalbardicum*, and from Northern Norway (10 individuals from 4 sites), putatively belonging to *A. brevicorne.* As recommended by Chen et al. [29], we used the combination of *gnd* + COI as the aphid barcode, which has been proven to be a good tool for resolving aphid taxonomy ambiguities. We amplified a 900bp fragment of the *gnd* gene, which encodes the third enzyme of the pentose phosphate pathway, 6-phosphogluconate dehydrogenase (6PGD) and situated on the genome of *Buchnera,* the aphid primary symbiont. In parallel, we amplified a 660bp fragment of the cytochrome oxidase subunit 1 gene (COI) located on the aphid mitochondrial genome. Primer sequences and PCR amplification conditions were as described in [29]. Both strands for each overlapping fragment were assembled using the sequence-editing software Bioedit 5.0.9 [30]. DNA Sequences were aligned using ClustalW 1.81 default settings [31]. Alignments were translated to amino acids using MEGA version 4 [32] to detect frameshift mutations and premature stop codons, which may indicate the presence of pseudogenes. We calculated sequence divergences using a K2P distance model in MEGA version 4. A phylogenetic tree was built for each gene separately, as well as on concatenated sequences of both loci. Sequences were deposited in Genbank under the accession numbers MN718201-MN718214.

## 3. Results

### 3.1. Overall Patterns of Geographic Distribution and Population Differentiation

Populations of *A. svalbardicum* were common but heterogeneously distributed along the west coast of Spitsbergen. This species was recorded up to 79°12’N in Krossfjorden. No population was found beyond this latitude, which may well represent the northernmost location for any aphid. Surveys resulted in the collection of 920 *A. svalbardicum* individuals from 29 sites from Spitsbergen and 180 *A. brevicorne* individuals from 9 sites from Lapland. Genotyping failures were very low (less than 2%), so that 1083 individuals with missing data at no more than one locus were retained for genetic analyses.

By considering both aphid species, overall F_ST_ coefficient was 0.280 and only 6 out the 570 (1%) pairwise comparison tests for genetic differentiation were not significant at α = 0.1, indicating a strong genetic structure among populations (see Figure S1). These non-significant pairwise F_ST_ values included either some populations separated by less than 100 metres or by different time intervals. Temporal changes in genetic structure were limited compared to spatial variation: considering aphid populations sampled for different time intervals, F_ST_ coefficients generally did not exceed 10%. The notable exception was for *A. svalbardicum* Camp Zoé population where 42% differentiation was found between 2004 and 2009 samples. Genetic differentiation was marked between populations from *A. brevicorne* and *A. svalbardicum* (F_ST_ = 0.219), with F_ST_ values ranging from 0.26 to 0.58, but also for *A. svalbardicum* Krossfjorden populations vs. other *A. svalbardicum* populations, with F_ST_ values ranging from 0.18 to 0.68. Interestingly, populations of *A. svalbardicum* from Spitsbergen showed significantly higher genetic differentiation (mean pairwise comparison of F_ST_ = 0.256) than populations of *A. brevicorne* from Lapland (mean pairwise comparison of F_ST_ = 0.115) (ANOVA, F = 123.3, df = 2, *p* < 0.001).

**Figure 2:**
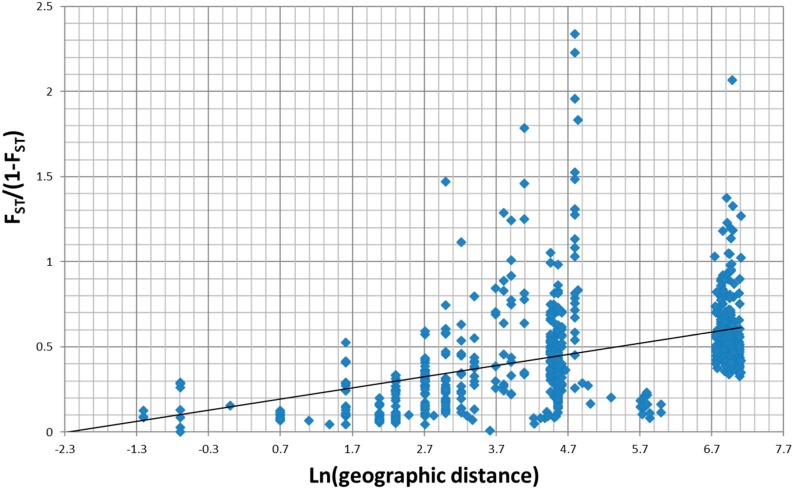
Correlation between genetic (F_ST_/(1- F_ST_) and geographic (kilometres in Ln scale) distances for samples of *A. svalbardicum* and *A. brevicorne.*

Isolation-by-distance analyses revealed a significant and positive correlation between genetic and geographic distance (one-sided *p* < 0.0001 from 10,000 randomizations, R² = 0.45) when all samples were considered (Figure 2). Correlation coefficients were even more pronounced when considering separately either all samples of *A. brevicorne* from Lapland (R² = 0.72), samples of *A. svalbardicum* from the Isfjorden zone (R² = 0.61) or from the Ny Ålesund zone (R² = 0.63).

### 3.2. Bayesian Analysis of Population Structure

**Figure 3:**
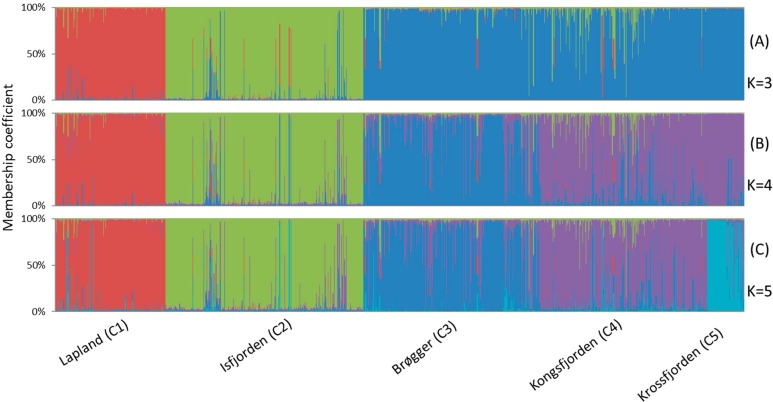
Results on the genetic assignment of individuals of *A. svalbardicum* and *A. brevicorne* based on the Bayesian method using the program STRUCTURE for K = 3 (**A**), K = 4 (**B**) and K = 5 (**C**). Each individual is represented by a column with its membership coefficient in each of the five clusters. Geographic origins of each genetic cluster are indicated on the bottom of the figure.

**Figure 4:**
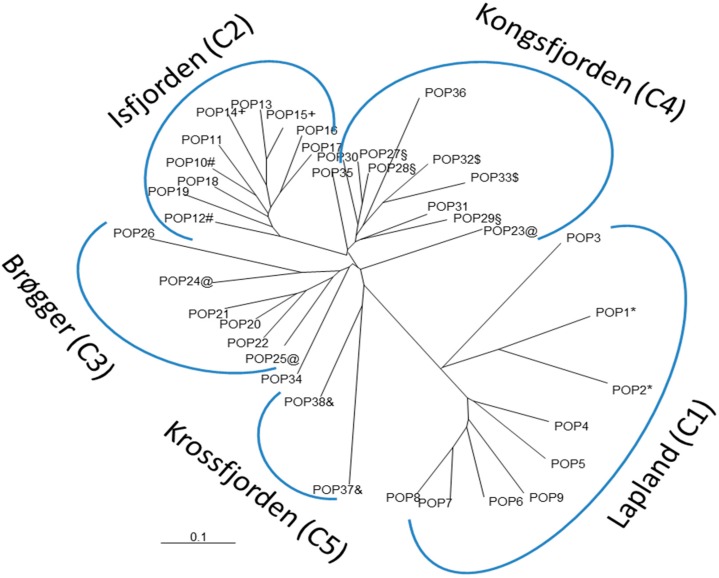
Neighbor-Joining tree constructed using allele shared distances between samples of *A. svalbardicum* and *A. brevicorne.* Populations with similar symbols were sampled at the approximately same site but at different time intervals.

STRUCTURE analyses were performed on all 1083 aphid individuals from the 38 samples (*A. svalbardicum* and *A. brevicorne* populations). Both log probabilities [Pr(X ǀ K)] and ΔK calculated on 10 iterations for K = 2 to K = 30 indicated an optimal clustering into three distinct groups which correspond to well-defined geographic zones. At K = 3 (Figure 3A), the first cluster (C1) included all *A. brevicorne* individuals from Lapland, the second cluster (C2) comprised most *A. svalbardicum* individuals from the Isfjorden zone, and the third cluster included most *A. svalbardicum* individuals from the Ny Ålesund zone. At K = 4 (Figure 3B), the two first clusters remained differentiated, while the third cluster was split into two genetic groups, one (C3) formed with *A. svalbardicum* individuals from the south side of Kongsfjorden (Brøgger peninsula), another (C4) formed with *A. svalbardicum* individuals from the north side of Kongsfjorden (including islands scattered in the fjord). At K = 5 (Figure 3C), most individuals from the two northernmost sites located in the Krossfjorden area differentiated from cluster C4 and formed a new cluster (C5). Pairwise comparisons of F_ST_ values among the five clusters showed that C2 and C5 were the most differentiated, while C3 and C4 were the closest genetically (Table 3). The analysis of genetic distances computed between the 38 aphid samples confirmed this differentiation pattern in five main groups, with *A. brevicorne* populations from Lapland clearly separated from *A. svalbardicum* populations from Spitsbergen (Figure 4).

### 3.3. Within-Population Structure

Populations of *A. svalbardicum* from Spitsbergen generally showed lower gene diversities compared with *A. brevicorne* populations from Lapland (Table 4). Mean expected heterozygosity and allele richness differed significantly between both aphid species (Hexp: LMM, F = 6.13, df = 1, *p* = 0.028; Allelic richness: LMM, F = 12.14, df = 1, *p* = 0.004): mean expected heterozygosity and allele richness were 0.515 and 3.87, respectively for *A. brevicorne* populations from Lapland vs. 0.430 and 3.04 for *A. svalbardicum* populations from Spitsbergen. The combination of the eight microsatellite loci allowed the discrimination of a large number of multilocus genotypes (MLGs). Most populations had G/N ratios near or equal to 1, meaning that each individual bore a unique MLG (Table 3). The *A. svalbardicum* population from Camp Zoé 2004 (northernmost site sampled in 2004) was the only exception with a G/N of 0.30. This population also had the lowest expected heterozygosity and allele richness (0.164 and 1.75, respectively). Populations showed no heterozygote excess, while about a third of them showed a heterozygote deficit. As expected, populations in heterozygote deficit also had positive F_IS_ values, with the two *A. brevicorne* populations from Abisko (Sweden) showing the highest values (above 0.3). Again, *A. svalbardicum* population from Camp Zoé 2004 was special in that it displayed a very high negative F_IS_ value (−0.281). Overall, F_IS_ values did not vary between the two aphid species (LMM, F = 1.67, df = 1, *p* = 0.217). Linkage disequilibria were frequent among pairs of loci, ranging from 5% to 43% of the tests, and the extent of linkage disequilibrium did not differ according to aphid species (LMM, F = 0.33, df = 1, *p* = 0.572).

### 3.4. Within-Population Structure

**Figure 5:**
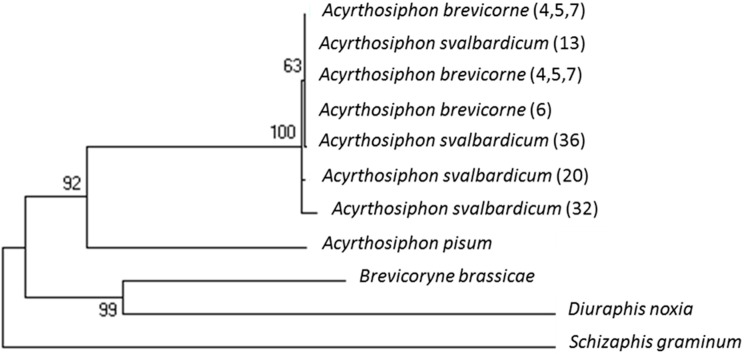
Phylogenetic tree built on concatenated sequences of COI and *gnd* genes amplified from *A. svalbardicum* and *A. brevicorne* samples. Numbers in bracket refer to sample origins, as coded in Table 1. Sequences of outgroups have been retrieved from Genbank.

The sequence analysis of COI fragment revealed 11 haplotypes among the samples from *A. brevicorne* and *A. svalbardicum*. However, the divergence between these haplotypes was very small and not associated with geographic origin. As a result, samples from *A. brevicorne* and *A. svalbardicum* grouped in the same clade, very distinct from the outgroups such as *A. pisum*. For *gnd* sequences, all samples from *A. brevicorne* and *A. svalbardicum* shared the same haplotype. Again, this haplotype was very divergent from closely related outgroups. Not surprisingly, when sequences from both COI and *gdn* were concatenated, samples from *A. brevicorne* and *A. svalbardicum* grouped into the same clade and were well differentiated from the related species *A. pisum*, as seen in Figure 5.

## 4. Discussion

### 4.1. Distribution and Range Limit of A. svalbardicum in Spitsbergen

From our field surveys in Svalbard, during which time we extensively sampled *A. svalbardicum* in the summer, we were able to show that this aphid was rather common but heterogeneously distributed along the west coast of Spitsbergen. At a large scale, some zones were free of aphids such as beyond certain latitudes, in isolated places such as some islands or for other reasons we could not test here, e.g., snow cover depth [33]. At a small scale, patches of *Dryas* unoccupied by aphids usually coexisted with occupied patches without obvious reasons. This heterogeneous distribution of the *A. svalbardicum* populations confirmed previous studies but does not allow us to substantiate the hypothesis that site occupancy increased with geographical distance from the fjord mouth [10]. From our study, we also showed that *A. svalbardicum* could not establish populations beyond 79°12′, which constitutes its northernmost limit and the most north location for any aphid. It is likely that beyond this latitude, prevailing climatic conditions prevent aphid populations from persisting and growing due to physiological constraints.

### 4.2. Divergence between A. svalbardicum and A. brevicorne

The Svalbard archipelago was completely covered by ice during the last glacial maximum, which occurred 15,000 years ago, except for some small mountain areas in the northwest [34]. Although some plants seem to have survived in situ in ice-free areas such as nunataks, uplands and dry coastal shelves [35,36], it has been shown that *Dryas octopetala*, the exclusive host plant for *A. svalbardicum* and *A. brevicorne,* colonised Svalbard from Russian after the last glaciation [37]. We may thus reasonably conclude that *A. svalbardicum* originated from postglacial dispersal either from North American or European refugia. The genetic analysis as here assessed using microsatellite markers, showed no marked increase in divergence between populations from *A. brevicorne* and from *A. svalbardicum*: the populations from Lapland were differentiated from those from Svalbard but differentiation coefficients (F_ST_) were not higher than between some distant Spitsbergen populations. In addition, analysis using two barcodes, which generally differentiate aphid taxa with good confidence [29,38], did not allow the separation of *A. brevicorne* and *A. svalbardicum.* Given the fact that the two species share *Dryas* as their exclusive host plant and differ only slightly in terms of their morphology [13,16], it is probable the two sister species split recently and certainly after the last glacial episode, 10,000–15,000 years ago. The relatively higher allelic richness in Lapland samples could reflect colonization events of Svalbard by *A. svalbardicum* from mainland Scandinavian populations, although this hypothesis needs further supports.

### 4.3. Signatures of Inbreeding in Populations of A. brevicorne and A. svalbardicum?

Populations of *A. brevicorne* and *A. svalbardicum* generally showed as many multilocus genotypes as sampled individuals (i.e., G/N generally equals 1). This means that the set of markers used had sufficient power to resolve most of the genotypic diversity present locally. In the rare cases where several individuals shared the same multilocus genotype, we may have sampled individuals belonging to the same genetic clone. This is possible for *A. brevicorne* populations because this species produces several clonal generations during its annual life-cycle [16]. Even for *A. svalbardicum,* which generally produces only two clonal generations a year, the probability of sampling two individuals from the same clone in the same patch is not zero. However, this could hardly explain the situation of Camp Zoé in 2004, where many individuals with the same MLGs have been found (Table 4). In this case, inbreeding between a few genetically individuals sound like a more likely explanation in such populations, which are at the northern limit of the species range.

Heterozygote deficit and linkage disequilibrium were frequent in populations of both *A. brevicorne* and *A. svalbardicum,* with no significant difference between the two species. This is highly suggestive of inbreeding, although other factors such as null alleles, selection and population subdivision might account for those patterns. Inbreeding is expected in small populations with limited dispersal between each other, which is obviously the case for *A. svalbardicum* (production of winged forms is exceptional in this species) and may also prevail in *A. brevicorne* (despite the possibility of this species producing winged forms in response to crowding). Whether *A. brevicorne* and *A. svalbardicum* populations suffer from inbreeding depression has yet to be investigated.

### 4.4. Strong Spatial Differences in Population Structure of A. svalbardicum

In comparison with *A. brevicorne*, *A. svalbardicum* populations showed much stronger spatial differentiation (more than twice as great), while temporal variation, when assessed, was generally modest in both aphid species. We found that populations of *A. svalbardicum* were highly differentiated even at distances around 100 metres. This marked spatial genetic structure is not surprising in such a species with limited dispersal capabilities and high extinction probabilities. From our genetic structure analysis, it appears that geographic distances as well as physical barriers such as fjords and glaciers contribute to the observed spatial pattern of genetic variation in *A. svalbardicum* populations. Isfjorden populations formed a single genetic cluster, suggesting they derived from the same colonization event and still maintain some connectivity. By way of contrast, populations from the Ny Ålesund zone split into three distinct genetic clusters, which seem primarily isolated by physical barriers rather than by geographic distances. Populations from the small islands of Kongsfjorden were genetically related to each other and with other samples from the north part of the fjord. It is therefore, possible that island colonization may have been achieved by the rare winged forms of *A. svalbardicum* that have been only found so far in the north side of Kongsfjorden [11,12]. Biological evidence to date suggests that the production of winged forms is a sporadic event, which is insufficient to provide effective widespread dispersal under appropriate spatial and temporal scales [11]. The genetic data presented here support this hypothesis. The two populations of *A. svalbardicum* from Krossfjorden, sampled at the northern limit of the species range, were strongly differentiated from the remaining *A. svalbardicum* populations, including the geographically closest ones (i.e., north of Kongsfjorden). This differentiation likely results from a founder effect (i.e., colonization of this area by a few migrants) followed by substantial inbreeding, as suggested above from within-population structure analysis.

## 5. Conclusions

Very few genetic studies have so far been performed on terrestrial animals inhabiting the arctic environment. In the present work, we were able to assess the influence of the high arctic conditions on the population’s genetic structure of *A. svalbardicum,* in comparison with its close related species *A. brevicorne* living in a relatively less harsh environment. Physiological constraints prevent aphid colonization beyond the range limit we detected for *A. svalbardicum*, although the temperature increase recorded in Svalbard in the last few decades might well favour population establishment further north [39]. At this range limit, populations showed low gene and genotypic diversities and high genetic divergence, which seemingly limits the adaptation of *A. svalbardicum* to extreme environment. Low dispersal apparently accounts for the overall strong genetic differentiation among populations, but again this could change if warming induces a sudden increase of wing morph production recently reported in Svalbard [11,12]. The colonization history of Svalbard by *A. svalbardicum* requires further in-depth studies involving phylogeographic analyses of arctic and alpine populations of *Acyrthosiphon* sp. feeding on *D. octopetala*. This would allow for better inference of the source origins of *A. svalbardicum* populations that have colonised Svalbard. Finally, more work is needed to elucidate the mechanisms underlying the adaptation to the artic environment in both *A. svalbardicum* and *A. brevicorne,* which has led to such peculiar life cycles and traits.

## Figures and Tables

**Table 1:** Locations of the populations of *A. svalbardicum* from Spitsbergen (Svalbard) and *A. brevicorne* from Lapland (Northern Norway and Sweden). All aphid populations were collected on *D. octopetala* plants.

Species (Zone)	Sample ID	Population	Area	Date	Latitude	Longitude
*A. brevicorne* (Lapland)	POP1	Abisko1	Abisko	29/06/2006	68°21′127	18°49′324
	POP2	Abisko2	Abisko	18/06/2010	68°21′128	18°49′325
	POP3	Gild_01	Gildetun	29/06/2012	69°54′174	21°35′969
	POP4	Ham_Kva_01	Kvalsund	01/07/2012	70°29′811	23°58′695
	POP5	Honn_01	Honninsvag	02/07/2012	70°59′793	25°58′246
	POP6	Lak_01	Trollholmsund	01/07/2012	70°16′914	25°06′776
	POP7	Lak_Bor_01	Borselv	03/07/2012	70°19′482	25°24′383
	POP8	Lak_NE_01	Lakselv-NE	03/07/2012	70°14′815	25°25′197
	POP9	Lak_Sarv_01	Sarvvesvuotna	01/07/2012	70°17′251	25°06′411
*A. svalbardicum* (Isfjorden)	POP10	Endalen1-04	Isfjorden	30/06/2004	78°11′269	15°45′938
	POP11	Endalen2-04	Isfjorden	30/06/2004	78°11′069	15°44′288
	POP12	Endalen09	Isfjorden	14/07/2009	78°11′131	15°45′939
	POP13	LYB1-04	Isfjorden	01/07/2004	78°12′995	15°36′631
	POP14	LYB2-04	Isfjorden	01/07/2004	78°12′995	15°36′631
	POP15	LYB09	Isfjorden	22/06/2009	78°12′995	15°36′631
	POP16	AdventS1	Isfjorden	03/07/2004	78°10′146	16°06′522
	POP17	AdventS2	Isfjorden	03/07/2004	78°10′026	16°06′412
	POP18	AdventN	Isfjorden	20/07/2005	78°15′702	15°40′153
	POP19	Colesdalen	Isfjorden	19/07/2005	78°04′602	15°15′092
*A. svalbardicum* (Ny Ålesund)	POP20	Gasebu	Brøgger	06/07/2009	78°54′590	12°04′338
	POP21	Midre	Brøgger	07/07/2004	78°54′573	12°04′339
	POP22	Corbel	Brøgger	18/07/2004	78°53′698	12°10′215
	POP23	Daerten04	Brøgger	13/07/2004	78°51′179	11°49′826
	POP24	Daerten06	Brøgger	09/07/2006	78°51′179	11°49′826
	POP25	Daerten09	Brøgger	03/07/2009	78°51′179	11°49′826
	POP26	Traudalen	Brøgger	15/07/2004	78°53′234	11°33′579
	POP27	Blomstrand04	Kongsfjorden	08/07/2004	78°57′801	12°02′768
	POP28	Blomstrand06	Kongsfjorden	11/07/2006	78°57′801	12°02′768
	POP29	Blomstrand09	Kongsfjorden	08/07/2009	78°57′801	12°02′768
	POP30	OssianE	Kongsfjorden	09/07/2004	78°55′599	12°20′623
	POP31	OssianW	Kongsfjorden	20/07/2004	78°55′599	12°27′413
	POP32	Gerdoya04	Kongsfjorden	24/07/2004	78°59′233	12°16′438
	POP33	Gerdoya09	Kongsfjorden	07/07/2009	78°59′233	12°16′438
	POP34	Storholmen	Kongsfjorden	06/07/2009	78°55′424	12°12′324
	POP35	Observatoire	Kongsfjorden	07/07/2009	78°55′526	12°16′387
	POP36	Kapp Guissez	Kongsfjorden	25/07/2004	79°05′374	11°47′319
	POP37	CampZoe04	Krossfjorden	21/07/2004	79°11′841	11°56′485
	POP38	CampZoe09	Krossfjorden	29/06/2009	79°11′841	11°56′485

**Table 2:** Features of the eight microsatellite loci isolated from *A. svalbardicum* and used for genetic structure assessment of populations of *A. svalbardicum* from Spitsbergen and *A. brevicorne* from Lapland, i.e., names of loci, forward and reverse sequences of primers for locus amplification, size range of alleles in base pairs, Nei’s estimations of heterozygosity (Hobs = observed heterozygosity, Hexp = expected heterozygosity).

Locus	Sequence Forward	Sequence Reverse	Size Range (bp)	Hobs	Hexp
AS23	GGCACTGCTCTCATTACGGT	TTTTTCTTCGTCATCCCTCG	125–190	0.648	0.715
AS24	GTCTGATGATGCGCTTGAAA	GAACCCAAACGAGGTGAAAA	155–190	0.593	0.571
AS26	AGTCCGGAGGATAACAACGA	TCACGACCGAACACCATAAA	155–205	0.415	0.458
AS29	CACCAAAAAGTCGGGGTAGA	GCCGTTGTTGAAGACTATTTCC	160–180	0.529	0.528
AS37	CGACGGGCGAGTACCTATTA	TTTCAAGTAAACCGCTTCGG	160–195	0.334	0.363
AS41	ATCTACCGCCACCACTTACG	TCGTCGAGATGCTATTGCTG	225–145	0.307	0.324
AS43	GAAAAACGAGAAAACGCGAC	AGTCCCTGATGCAAACAACC	250–305	0.162	0.244
AS45	TGAACCTGCTCAACAGCAAC	CCATGTCCTGACTCATCACG	255–300	0.430	0.443

**Table 3:** Pairwise comparisons of F_ST_ values between the five genetic clusters (C1 to C5) calculated using the program STRUCTURE. See text for the description of the five clusters.

Cluster	C1				
**C1**	0	**C2**			
**C2**	0.311	0	**C3**		
**C3**	0.226	0.196	0	**C4**	
**C4**	0.281	0.185	0.097	0	**C5**
**C5**	0.277	0.398	0.216	0.232	0

**Table 4:** Genetic structure of the 38 samples of *A. svalbardicum* and *A. brevicorne* from Svalbard (Spitsbergen) and Lapland (Northern Norway and Sweden), respectively: G/N, genotypic diversity measured by the ratio of multilocus genotypes found in the sample over the sample size; Hobs, observed heterozygosity; Hexp, expected heterozygosity; HetDef, test for heterozygote deficit under Hardy Weinberg equilibrium; HetXs, test for heterozygote excess under Hardy Weinberg equilibrium; F_IS_, inbreeding coefficient (asterisks indicate values significantly different from zero at 95% threshold); % LD, percentage of locus pairs in linkage disequilibrium.

Species/Zone	Sample ID	Size	G/N	Hobs	Hexp	Allele Richness	Hetdef (*p*-val)	HetXs (*p*-val)	F_IS_	% LD
*A. brevicorne* (Lapland)	POP1	20	0.94	0.402	0.650	5.375	0.0000	1.0000	0.405 *	25
	POP2	15	1.00	0.423	0.576	3.75	0.0000	1.0000	0.302 *	7
	POP3	9	1.00	0.453	0.409	2.75	0.3626	0.6374	−0.048	0
	POP4	19	1.00	0.449	0.486	4.125	0.0150	0.9850	0.108 *	7
	POP5	20	1.00	0.558	0.517	4.000	0.5875	0.4125	−0.050	10
	POP6	23	0.95	0.518	0.497	4.125	0.3210	0.6790	−0.018	7
	POP7	25	1.00	0.569	0.612	4.125	0.0152	0.9848	0.096	14
	POP8	23	1.00	0.581	0.554	4.375	0.6860	0.3140	−0.026	11
	POP9	22	0.74	0.438	0.486	3.625	0.0006	0.9994	0.122 *	57
*A. svalbardicum* (Isfjorden)	POP10	30	0.92	0.397	0.368	2.5	0.8958	0.1042	−0.063	0
	POP11	29	1.00	0.300	0.286	2.375	0.7518	0.2482	−0.027	0
	POP12	35	0.85	0.433	0.458	3.125	0.0783	0.9217	0.068	43
	POP13	28	0.82	0.432	0.394	2.75	0.5751	0.4249	−0.050	20
	POP14	30	1.00	0.427	0.407	3.000	0.4206	0.5794	−0.007	0
	POP15	23	0.96	0.399	0.407	3.375	0.0088	0.9912	0.028	29
	POP16	30	1.00	0.372	0.482	3.375	0.0000	1.0000	0.246 *	21
	POP17	30	1.00	0.432	0.445	3.125	0.0409	0.9591	0.048	5
	POP18	36	1.00	0.392	0.412	3.25	0.0023	0.9977	0.065	11
	POP19	42	0.80	0.341	0.336	2.875	0.6112	0.3888	−0.003	40
*A. svalbardicum* (Ny Ålesund)	POP20	30	1.00	0.430	0.470	3.000	0.0054	0.9946	0.102 *	18
	POP21	30	1.00	0.531	0.475	3.125	0.9796	0.0204	−0.102	14
	POP22	24	0.96	0.490	0.447	2.875	0.6763	0.3237	−0.073	4
	POP23	30	0.90	0.409	0.429	2.25	0.2237	0.7763	0.062	14
	POP24	33	0.97	0.440	0.435	3.125	0.6611	0.3389	0.004	11
	POP25	38	1.00	0.505	0.513	3.625	0.4422	0.5578	0.029	36
	POP26	30	0.92	0.395	0.342	2.000	0.5778	0.4222	−0.137	30
	POP27	47	1.00	0.512	0.487	3.5	0.1514	0.8486	−0.040	18
	POP28	48	1.00	0.494	0.507	3.875	0.0164	0.9836	0.035	11
	POP29	27	0.96	0.586	0.530	3.125	0.9412	0.0588	−0.087	19
	POP30	30	1.00	0.532	0.516	3.625	0.8227	0.1773	−0.015	11
	POP31	30	1.00	0.557	0.511	3.75	0.9257	0.0743	−0.073	7
	POP32	30	1.00	0.503	0.490	3.375	0.6773	0.3227	−0.009	7
	POP33	20	1.00	0.356	0.343	2.625	0.6057	0.3943	−0.012	10
	POP34	30	1.00	0.448	0.441	3.625	0.7376	0.2624	0.000	5
	POP35	29	1.00	0.478	0.559	3.5	0.0008	0.9992	0.162 *	11
	POP36	29	0.96	0.363	0.332	2.25	0.8856	0.1144	−0.078	20
	POP37	30	0.30	0.213	0.164	1.75	0.5554	0.4446	−0.281 *	20
	POP38	29	1.00	0.438	0.429	2.875	0.5461	0.4539	−0.004	29

## Supplementary Materials

Figure S1: Pairwise comparisons of F_ST_ values between the 38 samples of *A. brevicorne* (samples 1 to 9) and *A. svalbardicum* (10 to 38).
