# Supplementary material for: Life on the Edge: Ecological Genetics of a High Arctic Insect Species and Its Circumpolar Counterpart
Source: Insects. 2019 Nov 26;10(12):427. doi: 10.3390/insects10120427 (PMC6955800; doi:10.3390/insects10120427)
Supplement: Supplementary file 1 [file insects-10-00427-s001.pdf]

insects

MDPI

Supplementary Figure 1

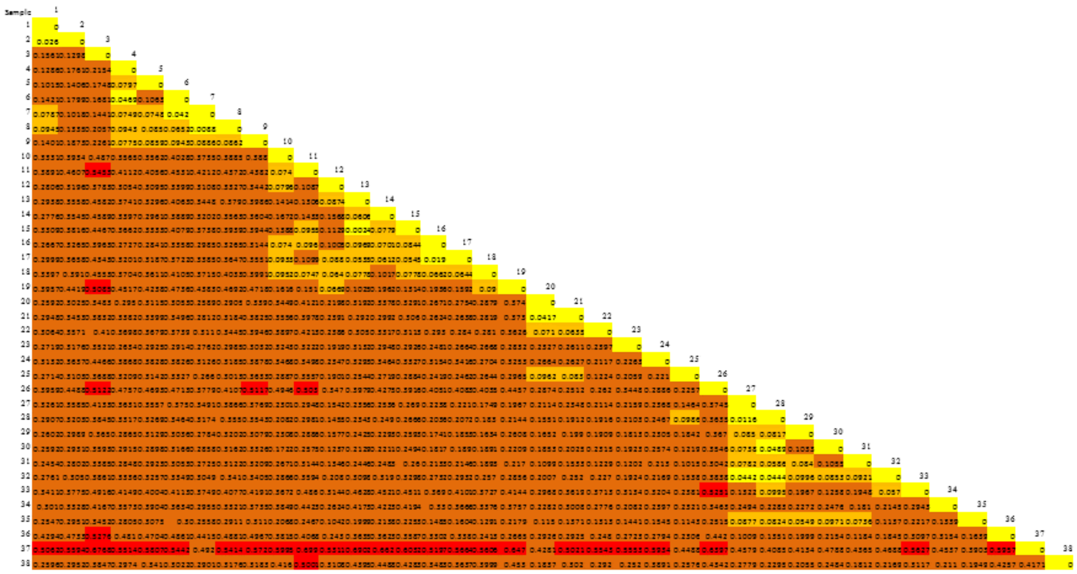

Figure S1. Pairwise comparisons of F_{ST} values between the 38 samples of *A. brevicorne* (samples 1 to 9) and *A. svalbardicum* (10 to 38).
